# The complete chloroplast genome sequence of the *Manglietia longirostrata* Sima, a rare and endemic species to china

**DOI:** 10.1080/23802359.2020.1791758

**Published:** 2020-07-15

**Authors:** Wei Luo, Zhuoran Li, Yongkang Sima, Tao Xu

**Affiliations:** a College of Ecology and Environmental Science, Yunnan University, Kunming, Yunnan, China; b Yunnan Academy of Forestry & Grassland Science (formerly Yunnan Academy of Forestry), Kunming, Yunnan, China

**Keywords:** Chloroplast genome, endemic species, *Manglietia longirostrata*, Magnoliaceae

## Abstract

*Manglietia longirostrata* Sima is a rare and endemic species in China. The complete chloroplast genome (cpDNA) of *M. longirostrata* was sequenced and assembled in this study. The cpDNA is 160,049 bps in length, contains a large single-copy region (LSC) of 88,098 bp and a small single-copy region (SSC) of 18,861 bp, separated by a pair of identical inverted repeat (IR) regions of 26,571 bp, each. The genome contains 123 genes, including 73 protein-coding genes, 8 ribosomal RNA genes, and 37 transfer RNA genes. Phylogenetic analysis of cp genome of *M. longirostrata* with 11 chloroplast genomes previously reported in the Magnoliaceae shows that *M. longirostrata* is close to *Manglietia megaphylla* with high bootstrap value.

*Manglietia longirostrata* (D. X. Li et R. Z. Zhou ex X. M. Hu, Q. W. Zeng et L. Fu) Sima was combined and stated by Sima Yongkang (Sima et al. 2016), Which was described as *Magnolia hookeri* var. *Longirostrata* for the long-beaked apex of the follicles and scanning electron micrographs of pollen grains by Hu X. M. & Q. W. Zeng, and they only found one individual in Maocaoping, Malipo County, Yunnan Province, China (Hu et al., 2012). After a careful field study, Sima Yongkang et al. found this species is scattered rarely in southeastern of Yunnan province in China, including Malipo, Yuanyang, and Jingpin county, the altitude range is 900 ∼ 1300 m, and the species was combined and stated in the genus *Manglietia* in the Magnoliaceae (Sima et al. 2016). Here, the annotated chloroplast (cp) genome sequence of *M. longirostrata* has been assembled and submitted to the GenBank with the accession number MT584886 and we performed a phylogenetic analysis which would benefit the genetic and phylogenetic research within this genus species.

The fresh leaves of *M. longirostrata* were collected from a tree cultivated in Kunming Arboretum, Yunnan Academy of Forestry & Grassland Science, Yunnan Province of China (25°9′5′′ N, 102°44′45′′ E). The sheets of the vouchered specimen (Sima and Lu 2012) are stored at the herbaria of YAF and YCP.

Total genomic DNA was extracted from the fresh leaves using Rapid Plant Genomic DNA Isolation Kit. The extracted DNA was sequenced using the Illumina Miseq platform (Illumina, San Diego, CA). In total, 85.1 M of 150-bp raw reads were retrieved. In order to ensure the quality of information analysis, the original reads must be filtered to get lean reads using Trimmomatic (Bolger et al. 2014). Sequencing data were assembled with SPAdes and GapFille (Boetzer and Pirovano 2012) was used to supplement the GAP of the contig obtained by stitching. The genome was automatically annotated using Prokka (Seemann 2014). OGDRAW v1.3.1 (Greiner et al. 2019) was used to generate a physical map of the cp genome.

The length of the complete cp genome sequence of *M. longirostrata* is 160,049 bp with four sub-regions: 88,098 bp of large single-copy (LSC) region and 18,864 bp of small single-copy (SSC) region are separated by two inverted repeats (IR) regions, each 26,571 bp, akin to other taxa in the family of Magnoliaceae (Liang et al. 2020). The overall CG content of the *M. longirostrata* cp genome is 39.30%. The cp genome contained 123 genes, including 73 protein-coding genes, 37 tRNA genes, and 8 rRNA genes. 15 genes (*trnA-UGC*, *trnE-UUC*, *trnK-UUU*, *trnS-CGA*, *trnL-UAA*, *trnC-ACA*, *trnE-UUC*, *rps16*, *rps12*, *rpl2*, *atpF*, *ndhB*, *ndhA*, *ycf1*, and *ycf3*) contain intron. The annotated genomic sequence was submitted to GenBank under the Accession Number of MT584886.

The complete cp genome of 9 reported Magnoliaceae species and one *Liriodendron* species as an outgroup were downloaded from NCBI GenBank and *Lirianthe delavayi’s* cp genome sequence from our work before (Liang et al. 2020) for the phylogenetic analysis. The combined datasets of 12 species were aligned by Kalign (Madeira et al. 2019). A maximum-likelihood (ML) tree was constructed in MEGA X with 1000 bootstrap replications (Kumar et al. 2018). The phylogenetic tree reveals that *M. longirostrata* is most closely related to *M. megaphylla* with strong bootstrap support (Figure 1) and the genera phylogenetic relationship is almost accord with the phylogenetic tree of *Lirianthe coco* (Sima et al. 2020) and all genera mentioned in this study are monophyletic under the taxonomical system of Magnoliaceae by Sima and Lu (2012). We think that the cp genome resource of *M. longirostrata* will be valuable for future studies in conservation genetics, taxonomy, phylogeny, and breeding in the *Manglietia* species.

**Figure 1.**
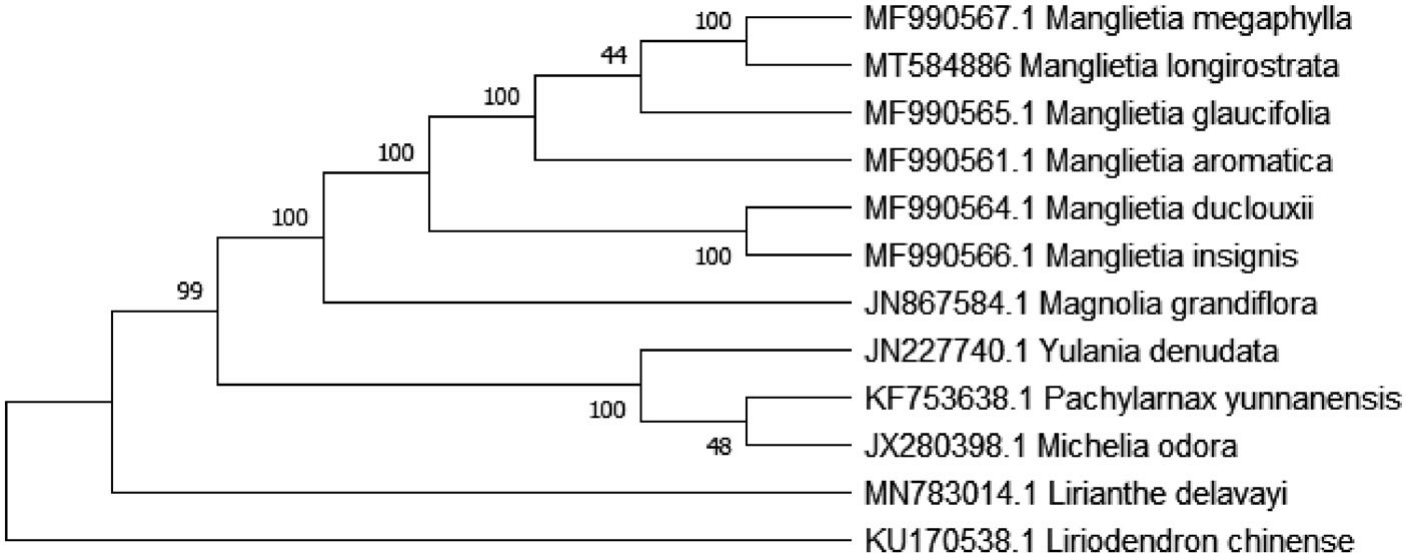
Phylogenetic tree using maximum-likelihood (ML) method based on the complete chloroplast genome of 12 species. Numbers above the node indicate bootstrap value.

## Data Availability

The cpDNA sequence of *Manglietia longirostrata* of this study are openly available in GenBank at https://www.ncbi.nlm.nih.gov/genbank/, reference number: MT584886. Scientific name of the organism in the paper: *Manglietia longirostrata* Sima. Geographic location of the specimen: Kunming Arboretum, Yunnan Academy of Forestry & Grassland Science, Yunnan Province of China (25°9′5′′ N,102°44′45′′ E).
